# Chromatographic Separation of *Breynia retusa* (Dennst.) Alston Bark, Fruit and Leaf Constituents from Bioactive Extracts

**DOI:** 10.3390/molecules25235537

**Published:** 2020-11-25

**Authors:** Stefano Dall’Acqua, Kouadio Ibrahime Sinan, Irene Ferrarese, Stefania Sut, Kouadio Bene, Mohamad Fawzi Mahomoodally, Nabeelah Bibi Sadeer, Gunes Ak, Gokhan Zengin

**Affiliations:** 1 Department of Pharmaceutical and Pharmacological Sciences, University of Padova, Via Marzolo 5, 35131 Padova, Italy; ire.ferrarese@gmail.com; 2 Department of Biology, Science Faculty, Selcuk University, 42130 Konya, Turkey; sinankouadio@gmail.com (K.I.S.); akguneselcuk@gmail.com (G.A.); 3 DAFNAE, Department of Agronomy, Food, Natural Resources, Animals and Environment, Agripolis Campus, University of Padova, 35020 Legnaro, Italy; stefania_sut@hotmail.it; 4 Laboratoire de Botanique et Phytothérapie, Unité de Formation et de Recherche Sciences de la Nature, 02 BP 801 Abidjan 02, Université Nangui Abrogoua, CI-YM. IV98 Abidjan, Ivory Cost; kouadio777@gmail.com; 5 Department of Health Sciences, Faculty of Medicine and Health Sciences, University of Mauritius, 80832 Réduit, Mauritius; nabeelah.sadeer1@umail.uom.ac.mu

**Keywords:** antioxidant, bioactive compounds, Cup Saucer plant, enzyme inhibitors, traditional medicine

## Abstract

*Breynia retusa* (Dennst.) Alston (also known as Cup Saucer plant) is a food plant with wide applications in traditional medicine, particularly in Ayurveda. Extracts obtained with four solvents (dichloromethane, methanol, ethyl acetate and water), from three plant parts, (fruit, leaf and bark) were obtained. Extracts were tested for total phenolic, flavonoid content and antioxidant activities using a battery of assays including 2,2-diphenyl-1-picrylhydrazyl (DPPH), 2,2-azino-bis(3-ethylbenzothiazoline-6-sulfonic acid) (ABTS), ferric reducing antioxidant power (FRAP), cupric reducing antioxidant capacity (CUPRAC), total antioxidant capacity (TAC) (phosphomolybdenum) and metal chelating. Enzyme inhibitory effects were investigated using acetylcholinesterase (AChE), butyrylcholinesterase (BChE), tyrosinase, α-amylase and α-glucosidase as target enzymes. Results showed that the methanolic bark extract exhibited significant radical scavenging activity (DPPH: 202.09 ± 0.15; ABTS: 490.12 ± 0.18 mg Trolox equivalent (TE)/g), reducing potential (FRAP: 325.86 ± 4.36: CUPRAC: 661.82 ± 0.40 mg TE/g) and possessed the highest TAC (3.33 ± 0.13 mmol TE/g). The methanolic extracts were subjected to LC-DAD-MS^n^ and NMR analysis. A two-column LC method was developed to separate constituents, allowing to identify and quantify forty-four and fifteen constituents in bark and fruits, respectively. Main compound in bark was epicatechin-3-*O*-sulphate and isolation of compound was performed to confirm its identity. Bark extract contained catechins, procyanidins, gallic acid derivatives and the sulfur containing spiroketal named breynins. Aerial parts mostly contained flavonoid glycosides. Considering the bioassays, the methanolic bark extract resulted a potent tyrosinase (152.79 ± 0.27 mg kojic acid equivalent/g), α-amylase (0.99 ± 0.01 mmol acarbose equivalent ACAE/g) and α-glucosidase (2.16 ± 0.01 mmol ACAE/g) inhibitor. In conclusion, methanol is able to extract the efficiently the phytoconstituents of *B. retusa* and the bark is the most valuable source of compounds.

## 1. Introduction

Since time immemorial, traditional medicines have been playing a significant role in the life of mankind and remain important for the modern medicine despite the abundant number of synthetic available drugs. Drug industries are heavily engaged in large-scale pharmacologic screening of medicinal herbs, spices and other food plants with the aim to develop efficient and safer medicines [1]. The screening of poorly explored medicinal plants can be a valuable source of new potential drug candidates. Phytochemicals can act as multiple target compounds and they can be valuable sources of health promoting compounds especially if we consider that the pathogenesis of many diseases is rather multi-factorial in nature, and not due to a single cause [2,3,4]. Furthermore, in recent years, nutraceuticals have been largely used and studied as health promoting products [5,6,7], and the search for new potential ingredients for such products, as well as the interest in deeper understanding of their mode of action and ability to interact with human health is today a hot topic.

Non-communicable diseases are on the rise causing 71% of deaths globally [8]. Such alarming situation has prompted the need to develop new drugs to improve quality of life and decrease preventable deaths. Alzheimer’s disease (AD) is a progressive neurodegenerative disorder with low acetylcholine level in the brain of patients. To date, scientists have failed to find a cure for AD due to the lack of understanding on the causes and mechanisms of the disease [9]. As a therapeutic strategy, cholinesterase inhibitors as donepezil, rivastigmine and galantamine are prescribed to patients to help postponing the symptoms of AD, but they present significant side effects [10,11].

Diabetes mellitus (DM) is chronic disease causing serious health problems [12] and most common treatment strategies comprise physical activity and proper diet. Pharmacologic treatments using metformin, acarbose, basal insulin or thiazolidinediones may delay or prevent the onset of DM but, similar to AD, these drugs possessed numerous side effects [13]. In recent years, different studies have considered traditional herbal medicines as possible option or complementary treatment. A strategy is to reduce dietary carbohydrate absorption by using natural inhibitors of enzymes. The target are α-amylase or α-glucosidase, such treatment can be valuable as inhibition reduce absorbable glucose reducing hyperglycaemia [14,15]. These compounds are considered due to their poor side effects [14].

The present study has considered different extracts of *Breynia Retusa* (Dennst.) Alston (*B. retusa*) for possible anti-cholinesterase and antidiabetic activity. Additionally, bearing in mind the complications related to DM, such as skin hyperpigmentation, the plant has also been investigated for its anti-tyrosinase property since tyrosinase enzyme is the main enzyme responsible to synthesize melanin [16].

Morphologically, *B. retusa,* also known as Cup Saucer plant, is a small tree or shrub growing to a height of 1–2 m with spread branches. It has broad, elliptical leaves which are 1.25–2.5 cm long. The male flowers are small, axillary and yellow. Fruits depressed-globose, 13–17 mm in diameter. It is widely distributed in Bangladesh, India, Sri Lanka, Vietnam, Malaysia, Thailand, Laos, China and Nepal [17]. The juice prepared from the leaves is used to cure inflammation, hyperglycaemia, diarrhoea, diuretic and relieve body pain. The fruits and twigs are used to treat dysentery and toothache, respectively [18]. Additionally, the stem can be used to treat conjunctivitis [17]. Available literatures showed that *B. retusa* yielded high level of phenolics and possessed antidiabetic and antioxidant effect [18,19].

Previous investigations on other *Breynia* species revealed the presence of unusual spiroketal sulfur containing glycosides named breinins [20,21,22,23]. Such compounds were isolated as active hypocholesterolemic agents from *B. officinalis* Hemsl. (*B. officinalis*) [20]. In other studies, ten different derivatives were isolated from the aerial parts of *B. fructicosa* (L.) Müll. Arg. (*B. fructicosa*), but in small amounts namely 10–150 mg each from 30 kg of plant material [24]. Literature indicated that up to now limited number of such derivatives have been reported. For instance, breynins A and B were isolated from *B. officinalis* (Koshiyama et al., 1976; Smith et al., 1992), and breynins B-D, G and epibreynins D-H were isolated from *B. fruticosa* [24]. From 10 kg of whole dried plant of *B. fructicosa*, isolated compounds were obtained in amount ranging from 40 to 620 mg. These compounds were then subjected to in vivo anti-inflammatory testing [25].

For these reasons, *Breynia* species appear to be attracting candidates to be considered as significant source of secondary metabolites with health promoting or medicinal properties. Thus, *B. retusa* extracts were screened in terms of antioxidant and enzyme inhibitory activity. Three plant parts namely leaf, fruit and bark were evaluated using a battery of in vitro bioassays to assess its potential biological activity. Furthermore, detailed phytochemical profiles were obtained via chromatographic approach using HPLC-DAD-MS^n^ analysis.

## 2. Results and Discussion

### 2.1. Bioactive Compounds

Plants are known to contain different classes of secondary metabolites in their various organs [26,27,28]. In this study, the phytoconstituents of the leaf, fruit and bark were extracted from different tissues of *B. retusa* using four solvents of different polarities (water > methanol > ethyl acetate > dichloromethane). The quali-quantitative composition of the extracts were analyzed and results are presented in Table 1.

Among the prepared extracts, the methanolic bark yielded the highest phenolic (145.79 ± 0.84 mg GAE/g) and flavanol (35.24 ± 0.39 mg CE/g) content while the methanolic leaf possessed the highest flavonoid content (34.49 ± 0.18 mg RE/g). However, a different trend was observed in another study. The ethyl acetate leaf and fruit extracts were found to be higher in flavonoid content (208.94 ± 50.03 and 259.33 ± 7.37 mg RE/g, respectively) and the methanolic leaf and fruit extracts yielded the highest phenolic content (81.05 ± 0.42 and 53.02 ± 0.95 mg GAE/g, respectively) [18]. Such difference in results can be explained by a number of parameters including growing conditions, temperature, geographical locations, seasonal variations, nutrients uptake and exposure to pollution [29]. Furthermore, it is important to highlight that plants and their respective ecosystems are connected whereby the type and amount of phytoconstituents are produced with respect to the plant’s environment and living conditions since secondary metabolites are only produced as part of the defensive mechanism of the plant [30]. Indeed, herein *B. retusa* was collected in Abidjan, Ivory Coast, while the sampling of the plant of the research group of Murugan was carried out in a different location viz., Tamil Nadu, India, thus explaining our results.

#### 2.1.1. Phytochemical Composition of Breynia Fruits, Leaves and Bark Extracts by NMR

**Figure 1:**
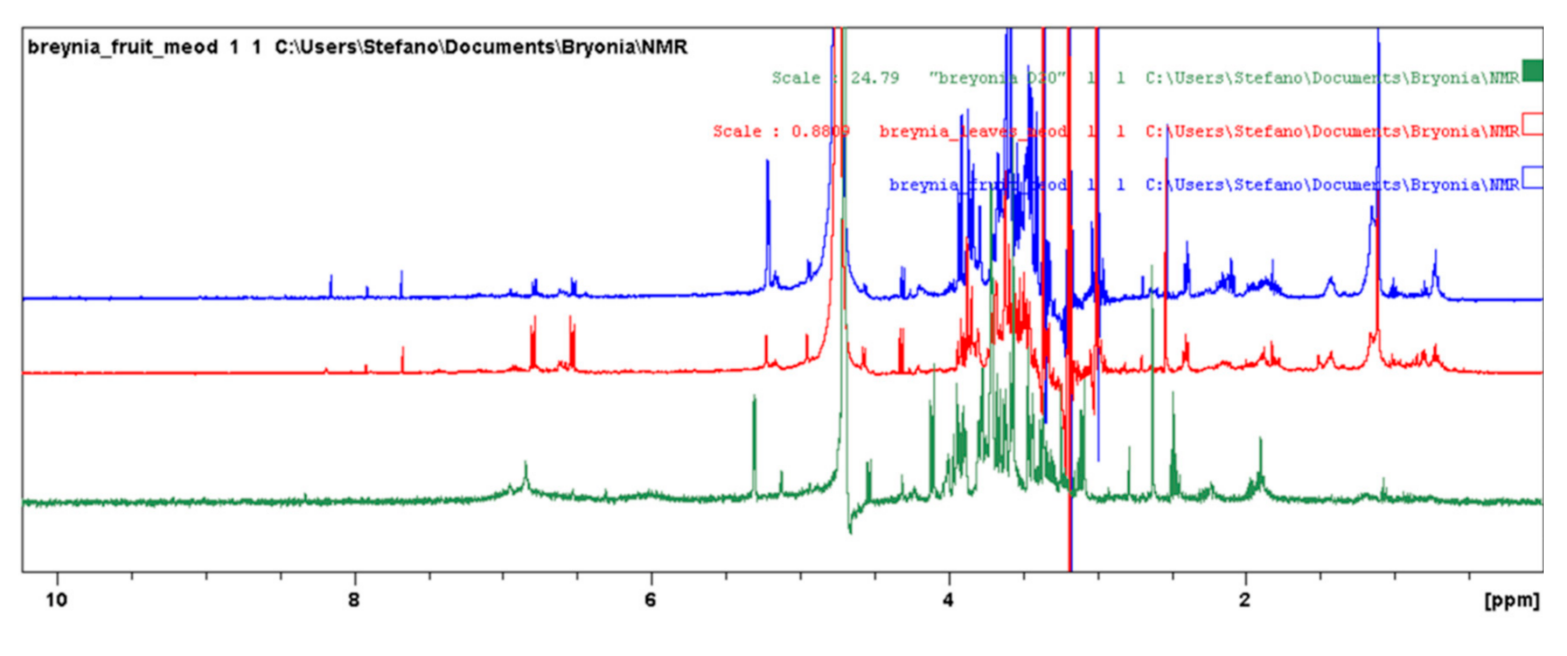
^1^H NMR of Deuterated methanol extract of *B. retusa* fruits in blue, deuterated methanol extract of *B. retusa* leaves in red, Deuterated water extract of *Breynia* in green.

The extracts were dissolved in deuterated chloroform and in spectra signals ascribable to fatty acid was observed in all the three samples. Then, the residual after chloroform extraction was collected, dried under vacuum and redissolved in deuterated methanol. Residuals of leaves and fruits were dissolved in methanol, and ^1^H NMR was reported in Figure 1 in blue and red, respectively. Due to the fact that bark residue was not significantly soluble in deuterated methanol, we decided to dissolve it in D_2_O and the spectra is showed in Figure 1 in green color. In all the ^1^H NMR spectra intense signal was present in the region between 3–4 ppm ascribable to sugar derivatives, that is confirmed by the presence of signals ascribable to anomeric proton of sugar moiety was present in the spectral region 4–5.5 ppm. In the spectra of leaves and fruits extracts, three singlets at δ 8.15, δ 7 91, δ 7.68 and two doublets at δ 6.78 and δ 6.55 (*J* = 8.94) were present suggesting the presence of aromatic compounds. In the spectra of bark there is a broad signal at δ 6.59–7.00, ascribable to proanthocyanidins or tannin derivatives. The screening obtained by NMR suggested that the bark extract is rich in tannin while aerial part and fruit extracts may contain other phenolics. To obtain more detailed information LC-DAD-MS^n^ approach was used.

#### 2.1.2. LC-DAD-MS^n^ of Bark Extract

The bark extract present complex composition and part of the work was dedicated to obtaining, method that allowed sufficient separation of the constituents.

**Figure 2:**
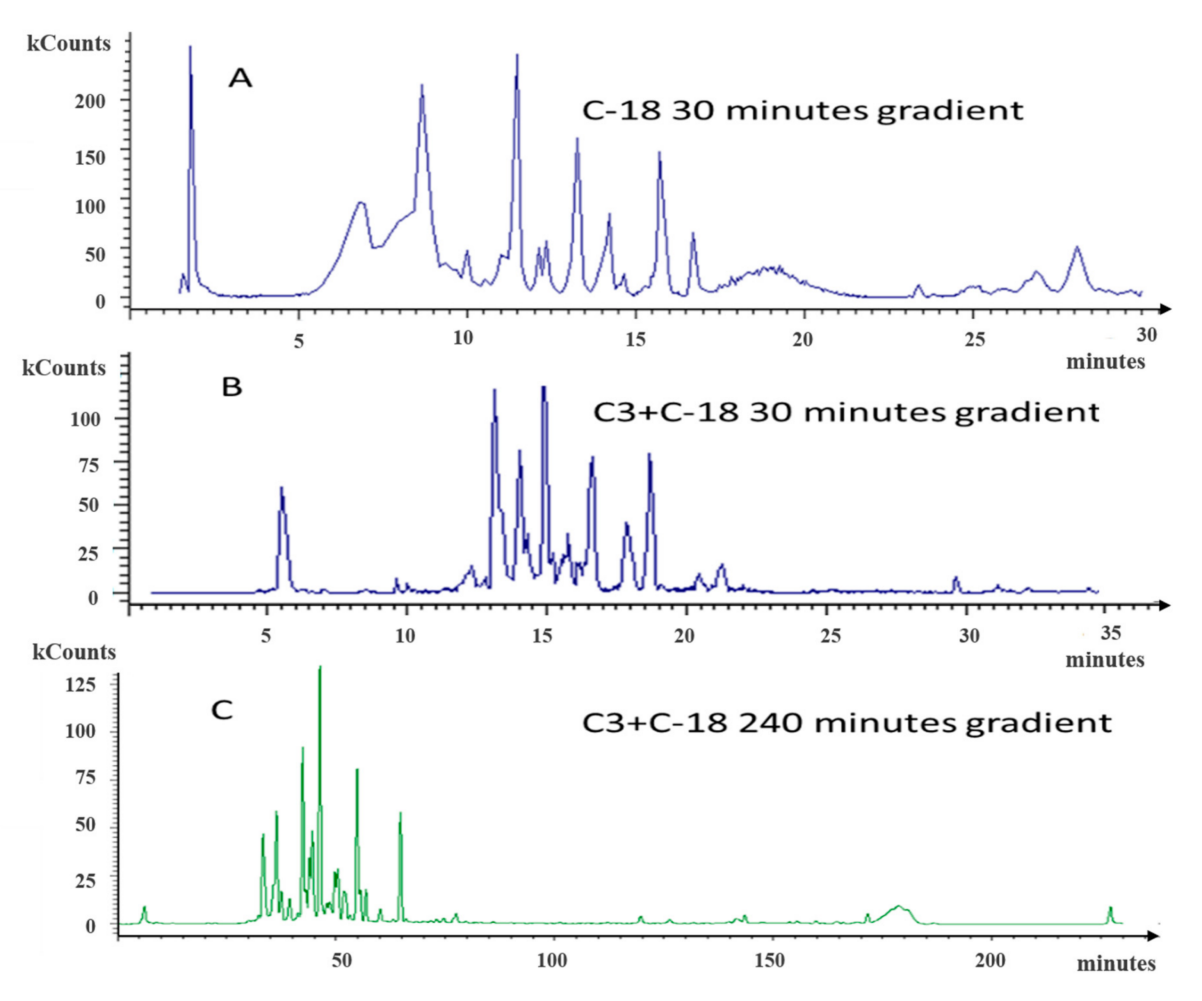
*B. retusa* bark LC-MS chromatogram peaks. Figures represent the spectra of the three different gradients employed, C-18 30 minutes’ gradient (**A**), C3 + C-18 30 minutes’ gradient (**B**), C3 + C-18 240 min gradient (**C**).

Figure 2 represent different chromatograms obtained during the method development. At beginning a reverse phase column (3.0 × 150 mm C-18 Eclipse XDB, Agilent, Santa Clara, CA, USA) was used and gradient was formed by aqueous formic acid and acetonitrile as reported in materials and methods. Many peaks appeared to be overlapped as visible in Figure 2 chromatogram A. Thus, to increase separation a second column was added. We selected a C-3 reverse phase (4.6 × 150 mm SB C-3 Agilent, Santa Clara, CA, USA) to obtain different selectivity but working with same eluent that was fitted before the C-18 column acting as a sort of pre-chromatography. Column diameter of 4.6 mm was selected in order to offer less resistance to flow and higher carbon loading due to complex matrix of the sample. Gradient elution was the same as used for chromatogram A. Figure 2B show the improvement of the separation with the use of the two columns. Then gradient was modified as indicated in materials and methods allowing more time for separation and result is reported in Figure 2C. This was selected as ideal conditions for all the samples.

**Figure 3:**
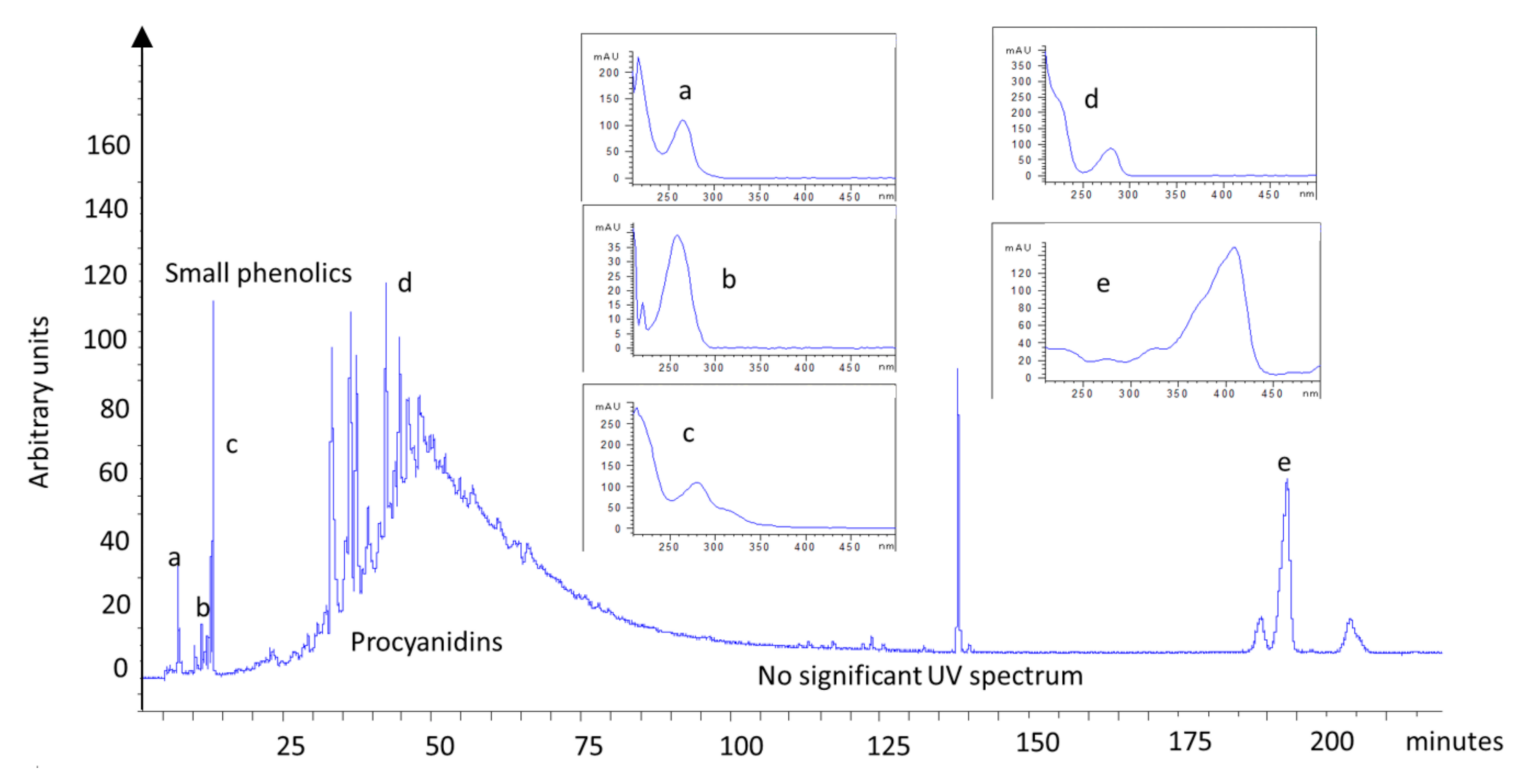
LC-DAD chromatogram of *B. retusa* bark extract. Superscript letters a,b,c,d indicate UV spectra of selected peaks. (**a**–**c**), UV spectra of small phenolics; (**d**), UV spectra of procyanidin; (**e**), UV spectra of unknown compound.

In the obtained conditions the LC-DAD of the bark sample showed numerous partially overlapping peaks with UV spectrum resuming catechin or procyanidins eluting in the chromatogram time 30–60 min. Peaks ascribable to small phenolics due to UV spectrum are revealed in the first part of the chromatogram as reported in Figure 3.

**Figure 4:**
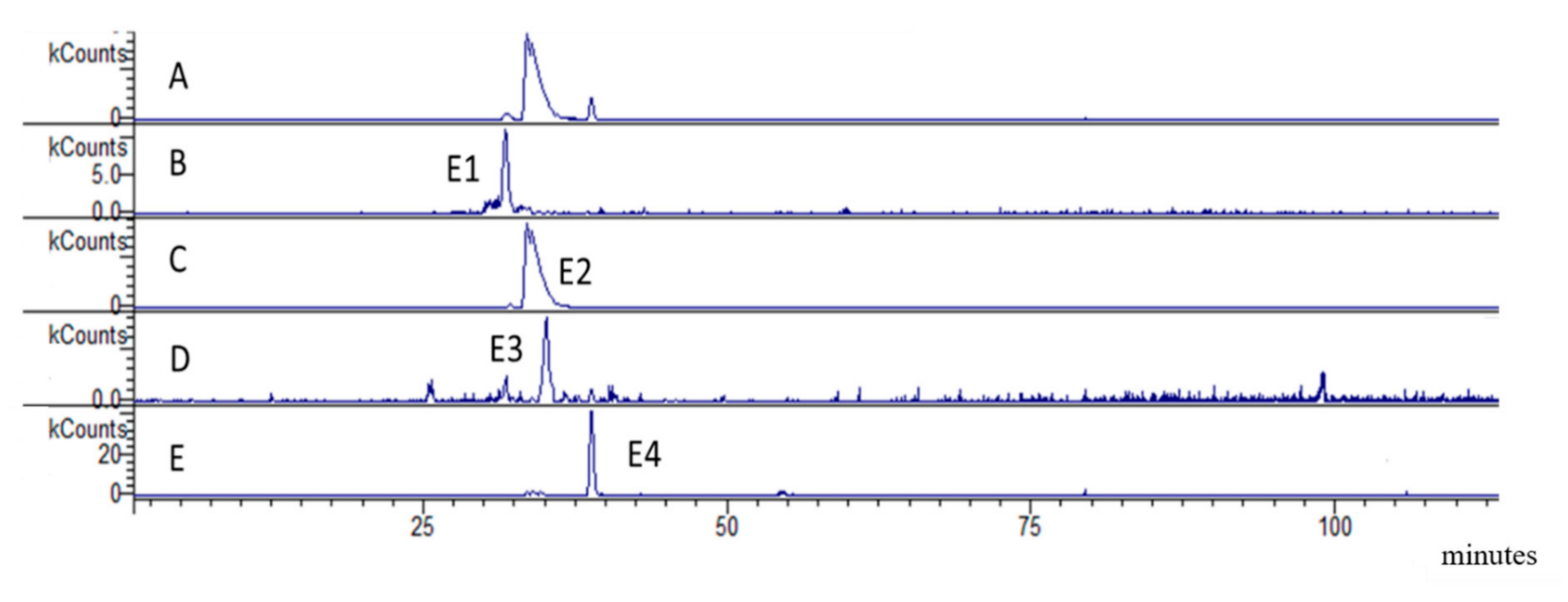
*B. retusa* bark, LC-MS chromatogram peaks, identified compounds are indicated as in Table 2. (**A**). total ion current of chromatogram; (**B**). epigallocatechin sulphate:E1; (**C**), Epicatechin-7-*O*-sulphate: E2; (**D**), Epigallocatechin: E3; (**E**), Epicatechin: E4.

**Figure 5:**
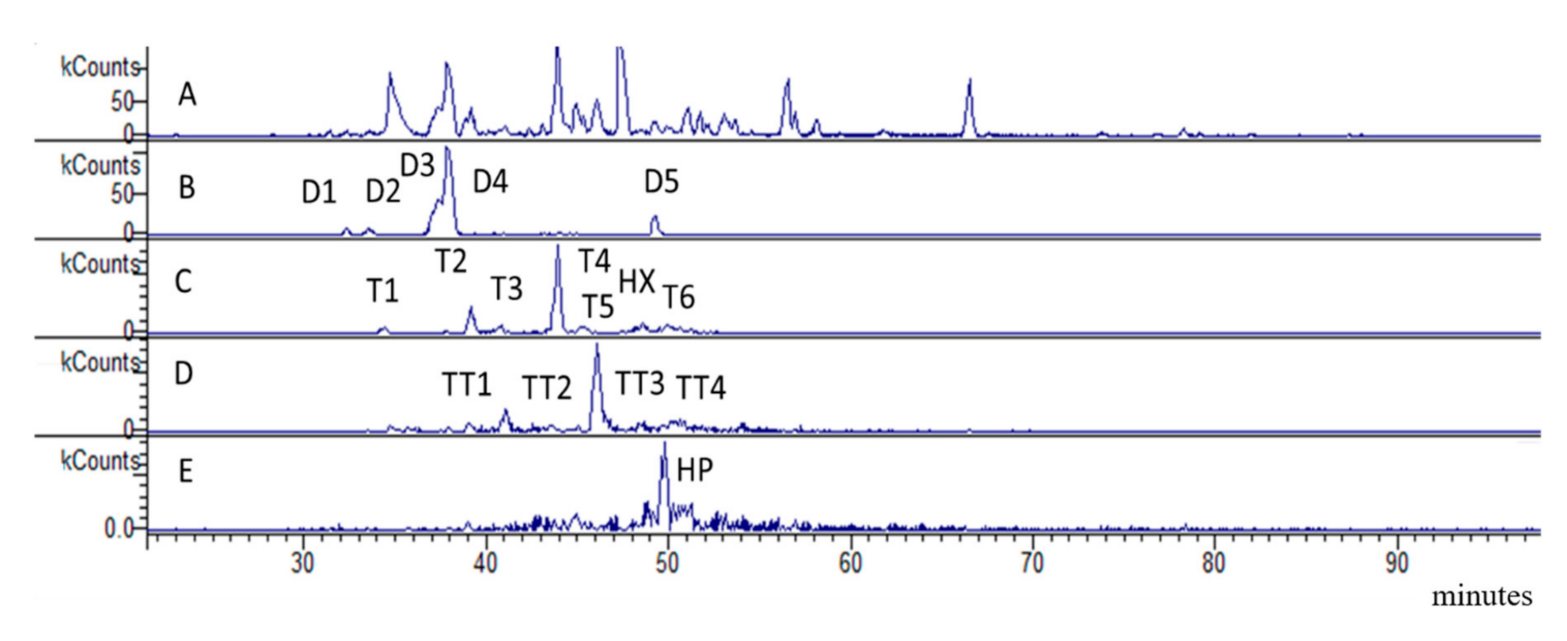
*B. retusa* bark, LC-MS chromatogram peaks, identified compounds are indicated as in Table 2. (**A**). total ion current of chromatogram; (**B**), proacyanidin dimers:D1–D5; (**C**), procyanidin trimers and hexamer: T1-T6, HX; (**D**), procyanidin tetramer: TT1-TT4; (**E**), procyanidin heptemer: HP.

**Figure 6:**
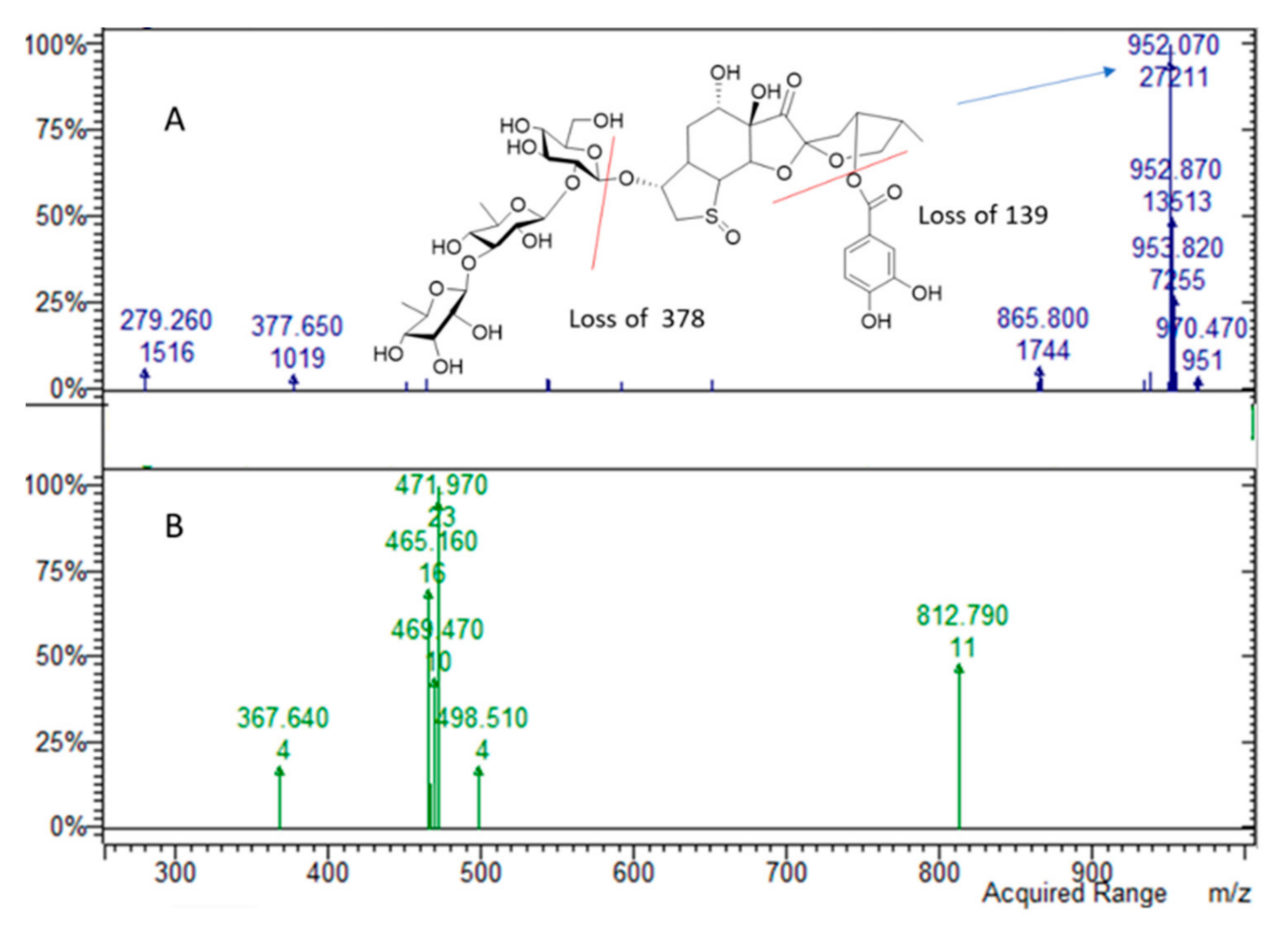
MS (blue spectrum) and MS^2^ (green spectrum) spectrum of peak at retention time 52 corresponding to the tentative assignment of Epibreinin B. (**A**), MS of ion 952 *m*/*z*; (**B**), MS^2^ of ion 952 *m*/*z.*

Due to MS^n^ data reported in Table 2, several peaks were identified as epicatechin and procyanidins derivatives. Epicatechin, epigallocatechin and PAC-B2 presence was confirmed by standard comparison. Their amounts were 4.9 mg/g, 0.46 mg/g and 13.1 mg/g for epicatechin, epigallotcatechin and PAC-B2, respectively. Other identified compounds on the basis of fragmentation and literature comparison are reported in Table 2 showing 29 identified constituents, and a chromatogram is shown in Figure 4 and Figure 5. Peak at 34.4 min was ascribable to epicatechin sulphate and represent the most abundant constituent (60.7 mg/g). Several derivatives with molecular ion [M − H]^−^ at *m*/*z* 577.5 and 1153.5 presenting typical MS^n^ fragmentation scheme ascribable to PAC-B are detected indicating the presence of dimeric and trimeric PAC-B. Poly-charged derivatives of PAC-B are also detected showing significant amounts of hexamers and heptamers. Small phenolic acids are also detected in particular protocatechuic, benzoic and gallic acid derivatives were identified and their total amount is 1.99 mg/g. The total amount of identified phenolics is 133.1 mg/g. Some other peaks were detected and four showed values of *m*/*z* as well as fragmentation that can resume the structure of some sulfur containing spiroketal derivatives previously isolated and identified from several *Breynia* species named with general indication of Breynins [20,21,22,23]. Our MS data allowed to speculate the presence of Epibreinin E, two epimeric derivatives of Breynin B and Breynin D, and a possible derivative of Breynin F (Table 2). An exemplificative MS and MS^2^ spectrum is reported in Figure 6 where the fragments due to the loss of the *p*-hydroxybenzoic acid and due to the loss of the terpenoid core are visible at *m*/*z* 812 and 471 respectively. Up to now there were no available breynin standards thus quantification was not possible. Furthermore, as reported in the literature the amount of such compounds is in general limited [24,25]; as previously reported, the isolation of such compounds, in general, yield 10–600 mg starting from 10 Kg of plant material, thus suggesting low abundance of these compounds in the plants.

**Chart 1:**
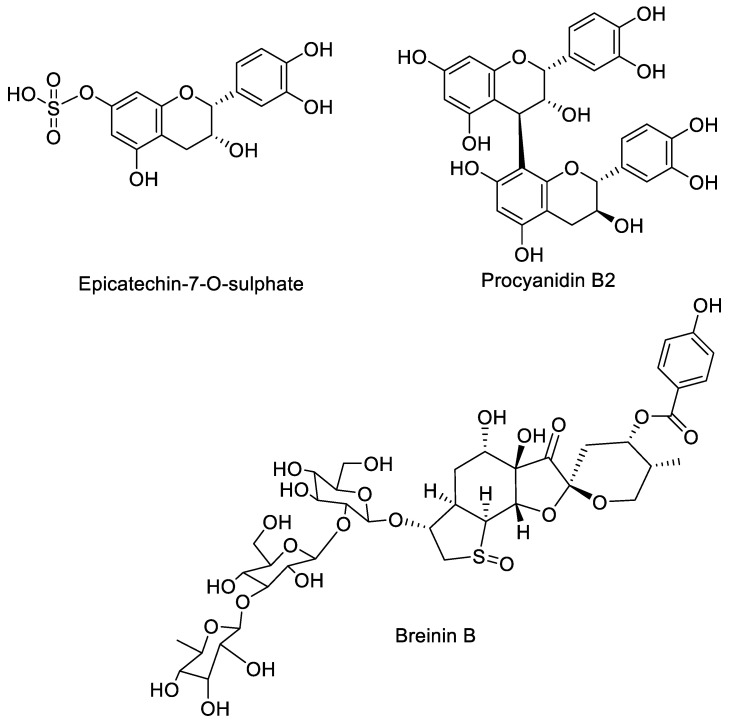
Main compounds identified in in *B. retusa* stem barks.

To confirm the structures of the most abundant compound we proceed with isolation. A total of 5 g of methanol extract of bark were applied to a sephadex column 4 × 100 cm and fraction were eluted with methanol. A total of 300 fractions of 20 mL were collected and pooled on the basis of their behaviour in TLC. From fraction 96–131 semipreparative HPLC yielded epicatechin-7-*O*-sulfate (50 mg). Structure was elucidated on the basis of NMR and MS data. The NMR assignment for the compound were in agreement with the literature [31] in particular the strong shift of the H-6 and H-8 values suggest that the sulphate group is linked to the 7-OH. The overall phytochemical constituents of the bark extract are reported in Table 2, chromatograms with identified compounds are reported in Figure 4 and Figure 5. Amount of phenolics in the bark extract is significant being more than 120 mg/g. Chart 1 report the most abundant compounds found in bark.

#### 2.1.3. Breynia Fruits and Leaves

**Chart 2:**
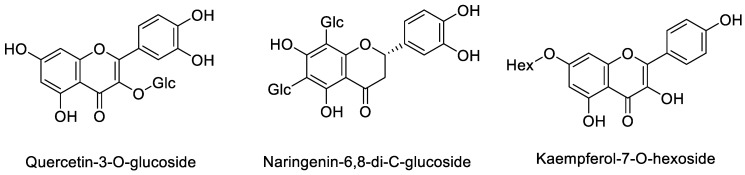
Main compounds identified in in *B. retusa* leaves and fruits.

**Figure 7:**
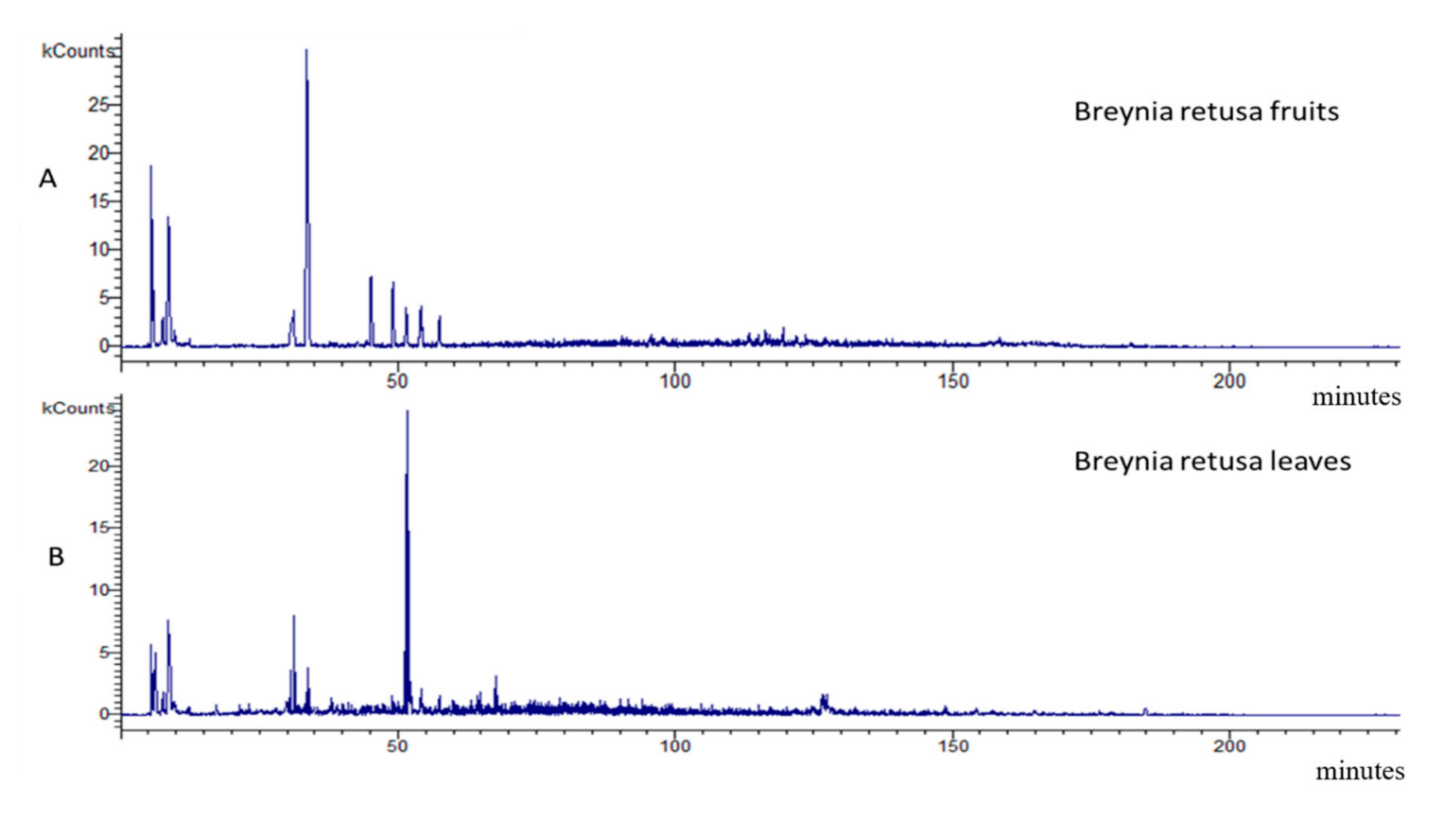
*B. retusa* fruit and leaves LC-MS Base Peak Ion (BPI) chromatograms. (**A**), Chromatogram of fruits; (**B**), Chromatogram of leaves.

LC-DAD-MS^n^ of *Breynia* fruits extract present different behavior compared to barks as shown in Figure 7. Peaks ascribable to catechin and/or procyanidins are almost not detectable. Intense peaks in the first part of the chromatogram are detected showing absorption maximum at 280 nm, some can be ascribed to phenolics. Chart 2 report the most abundant compounds found in fruits and leaves.

Leaves and fruits chromatograms are quite similar and the identified compounds are reported in Table 3, no peaks ascribable to procyanidins are detected, main constituents of fruits as observed in NMR are disaccharides that were also detected in LC-MS, but not quantified. Considering the phenolic compounds most abundant constituents are gallic acid and quercetin derivatives. Leaves and fruits contain also flavonoid glycosides that are absent in the barks sample (Table 3). Total amount of phenolics in leaves and fruits are 51 and 32 mg/g, respectively.

### 2.2. Antioxidant Activities

In order to understand the possible different antioxidant mechanisms of *B. retusa,* a set of assays were conducted. For example, DPPH and ABTS assays were used to assess radical quenching ability, FRAP and CUPRAC assays to investigate reducing power, metal chelating to determine ability of extracts to chelate ferrous ions and phosphomolybdenum to determine total antioxidant capacity. The results are presented in Table 4.

Overall, findings showed that there is a good correlation between the different assays except with metal chelating assay. Such kind of correlation was also observed in several other studies [32,33,34,35]. The methanol bark extract exhibited the highest antioxidant property with all assays except metal chelating. For instance, the latter extract displayed the most potent DPPH and ABTS radical scavenging activities (202.09 ± 0.15 and 490.12 ± 0.18 mg TE/g, respectively), strongest Fe^3+^ and Cu^2+^ reducing potential (325.86 ± 4.36 and 661.82 ± 0.40 mg TE/g, respectively) and possessed the highest total antioxidant capacity (3.33 ± 0.13 mmol TE/g). Scientists have claimed that highly polar solvents, especially methanol, have a high effectiveness as antioxidants [26], hence supporting our results. However, our findings are not in line with the study conducted by Murugan, Prabu, Chandran, Sajeesh, Iniyavan and Parimelazhagan [18], since a different plant part demonstrated high radical scavenging activity. For instance, the methanolic and ethyl acetate leaf extract displayed the highest DPPH and ABTS activity, respectively, (DPPH: IC50 = 2.42 µg/mL; ABTS: 7195.45 ± 44.31 µM TEAC/g).

Our results are consistent with the phytochemical composition results shown in Table 2 presenting methanolic bark extract with the highest phenolic content. Several lines of evidence have showed that plant extracts with high phenolic content exhibited substantially high antioxidant activity [36,37]. On the other hand, the dichloromethane fruit extract showed poor antioxidant activity with ABTS, FRAP and CUPRAC tests and inactivity was reported with DPPH and metal chelating assays. Interestingly, despite the fruit showed high nutritional composition in terms of starch, protein, amino acid and mineral content in the work of Murugan, Prabu, Chandran, Sajeesh, Iniyavan and Parimelazhagan [18], the results presented herein showed that the fruit possessed low phytoantioxidants content.

### 2.3. Enzyme Inhibitory Effects

In this research work, the extracts of *B. retusa* were screened for possible enzyme inhibitory effects against several non-communicable diseases including diabetes mellitus II (α-amylase and α-glucosidase), Alzheimer’s disease [acetyl- (AChE) and butyryl-cholinesterase (BChE)] and skin hyperpigmentation (tyrosinase). As presented in Table 5, the methanolic bark extract showing the highest anti-tyrosinase activity (152.79 ± 0.27 mg KAE/g) also showed the most potent anti-amylase (0.99 ± 0.01 mmol ACAE/g). Additionally, the three extracts, i.e., methanolic and water bark and methanolic fruit extracts were more active glucosidase inhibitor (2.16 ± 0.01; 2.14± and 2.14± mmol ACAE/g). However, no direct correlation was observed between the two cholinesterase enzymes. The ethyl acetate leaf extract showed the highest anti-AChE activity (5.13 ± 0.11 mg GALAE/g) but the same extract exhibited poor inhibition activity against BChE (2.25 ± 0.62 mg GALAE/g). In fact, the highest anti-BChE activity was recorded by the ethyl acetate fruit extracts.

Our results showed that the methanolic bark extract of *B. retusa* has strong inhibitory effects on α-amylase and α-glucosidase enzymes which may delay the degradation of starch and oligosaccharides. These findings are in agreement with the study of Murugan, Prabu, Chandran, Sajeesh, Iniyavan and Parimelazhagan [18]. Our data suggested that this potent activity may be attributed to the high level of phenolic present in the extract because phenolics are important bioactive compounds responsible for a broad spectrum of biological activities, including antidiabetic, anti-tyrosinase, antimicrobial, anti-inflammatory and anti-aging, among others [38].

On the other hand, despite several publications have suggested that flavonoids have strong inhibitory effects against amylase and glucosidase [39], our data do not corroborate with this fact. The methanolic leaf extract possessing the highest level of flavonoids (34.49 ± 0.18 mg RE/g) does not exhibit significant anti-amylase and anti-glucosidase activities. Instead, the methanolic bark extract yielding the highest level of flavanol showed potent antidiabetic activity. In fact, these results can be explained by the different classes of flavonoids present in the extracts. Indeed, it is reported that the number and configuration of hydroxyl groups play a crucial role in regulating the bioactivity of flavonoids [40]. Therefore, considering these aforementioned facts retrieved from literature, it can be said that the high anti-tyrosinase and antidiabetic activity exhibited by the methanolic bark extract may be associated to the abundant presence of both phenolic and flavanol content.

Irrespective of the type of plant parts assessed, the aqueous extracts were the least effective extracts in inhibiting the activity of enzymes. For instance, aqueous leaf extract poorly inhibited the activity of AChE (0.09 ± 0.01 mg GALAE/g) and α-amylase (0.19 ± 0.01 mmol ACAE/g) and no activity was reported for BChE while aqueous fruit extract was less successful in inhibiting tyrosinase (52.44 ± 3.87 mg KAE/g) and α-glucosidase (not active) enzymes (Table 5). These results may be attributed to the presence of low phenolic and flavonoid content.

### 2.4. Multiple Statistical Data Mining

In this work, unsupervised multivariate was also achieved in order to bring out pertinent information and so to improve interpretation of all results simultaneously, which are not always discernible in univariate analysis approach. In fact, the statistical processing of heterogeneous data using univariate statistic do not permit to display the clustering of samples with homogeneous special character. Therefore, principal component analysis (PCA) was firstly done to highlight the intrinsic similarities/dissimilarities between the samples based upon their pharmaceutical characteristics. PCA is a hugely popular well-established dimensionality reduction method that aims to find a low-dimensional subspace that synthesize, as much as possible, the total variation in the data.

**Figure 8:**
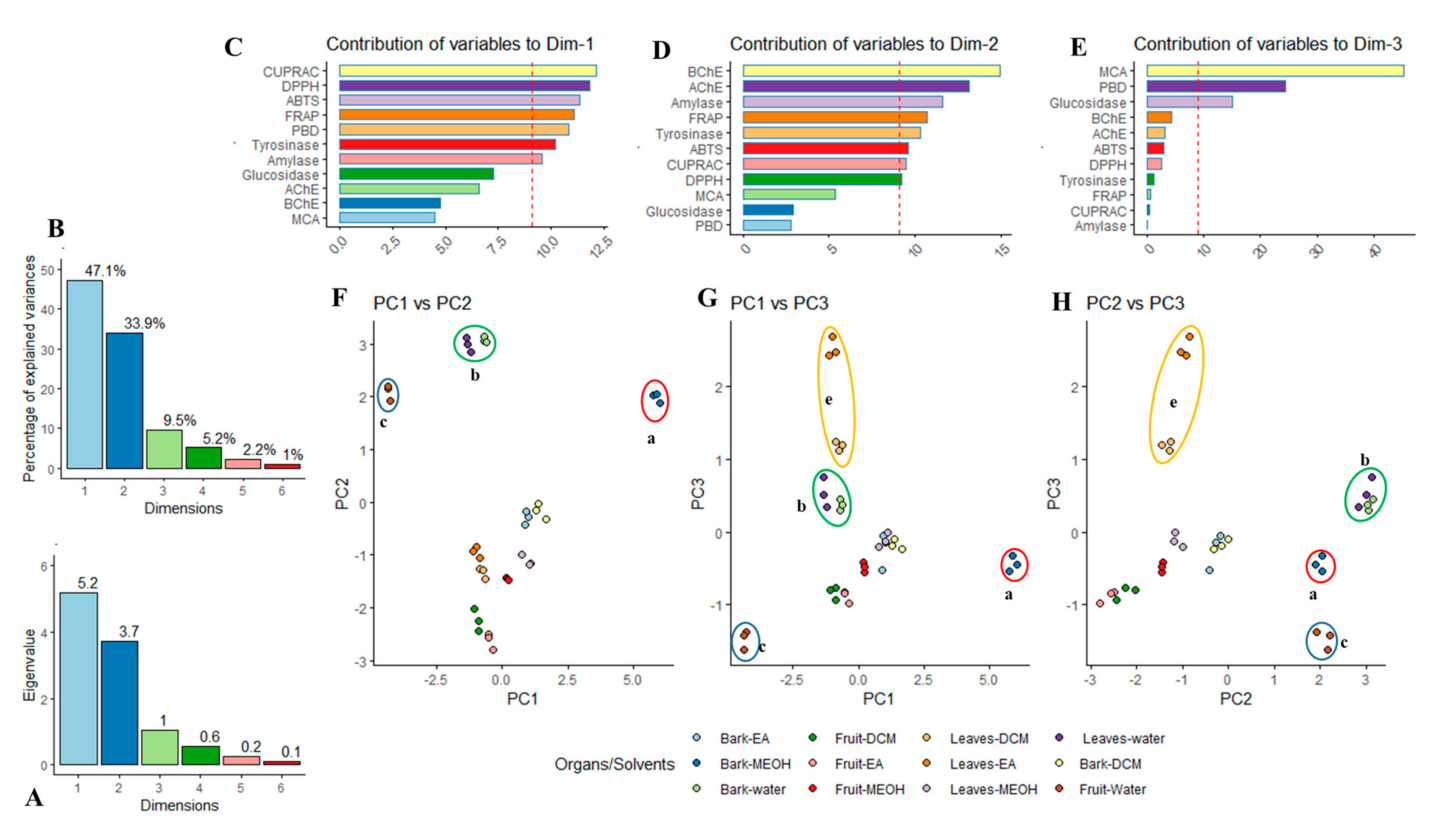
Unsupervised principal component analysis. (**A**,**B**), Scree plot of the eigenvalue and the percent of variance explained by each component; (**C**–**E**), Contribution of biological assays to each component; (**F**–**H**), Projection of the samples on the 1st, 2nd and 3rd factor planes (the dot (a–e) in Figure F–H indicates the groups in the tested extracts).

The results of PCA were depicted in Figure 8. An estimated 90.5% of the total variation was summarized by the first three principal components, which were found to have an eigenvalue higher than 1 (Figure 8). Thus, 90.5% of the information from the 11 initial variables (biological activities) can be condensed into relatively three new variables (principal components), which was significant by retaining most information. Figure 8 displayed that the most important variable contributing to PC1were the antioxidant assays and to a lesser extent the tyrosinase and amylase assays. Thus, PC1 is defined by the antioxidant properties and inhibition ability against tyrosinase and amylase. PC2 was described predominantly by BChE, AChE, amylase and modestly by FRAP and tyrosinase, whereas PC3 was bound to MCA, PBD and glucosidase. Therefore, PC1 can be expressed as the antioxidant power, PC2 was defined as the suppression potency against cholinesterase enzymes while PC3 was considered as Fe^2+^ chelating ability, the antioxidant level and inhibition ability against glucosidase enzyme. Figure 8C displayed the projection of the samples in the space of the first three retained principal components. It can be seen a dispersion of samples in the different score plots, with however the emergence of some groups. Indeed, by observing the three score plots, the methanol bark extract (group A) and water fruit extract (group C), respectively, were outstanding from the other extracts. In regard to both score plots, PC1 vs. PC2 and PC2 vs. PC3, the water extract of barks and leaves (group B), much closed together, appeared to be in a unique group, separated from the others. In addition, in the score plot PC1 vs. PC2, the eight remaining samples seemed not-to-distant; however, by viewing the score plots PC1 vs. PC3 and PC2 vs. PC3, we can notice the separation of DCM and EA extracts of leaves (E) of this large group.

**Figure 9:**
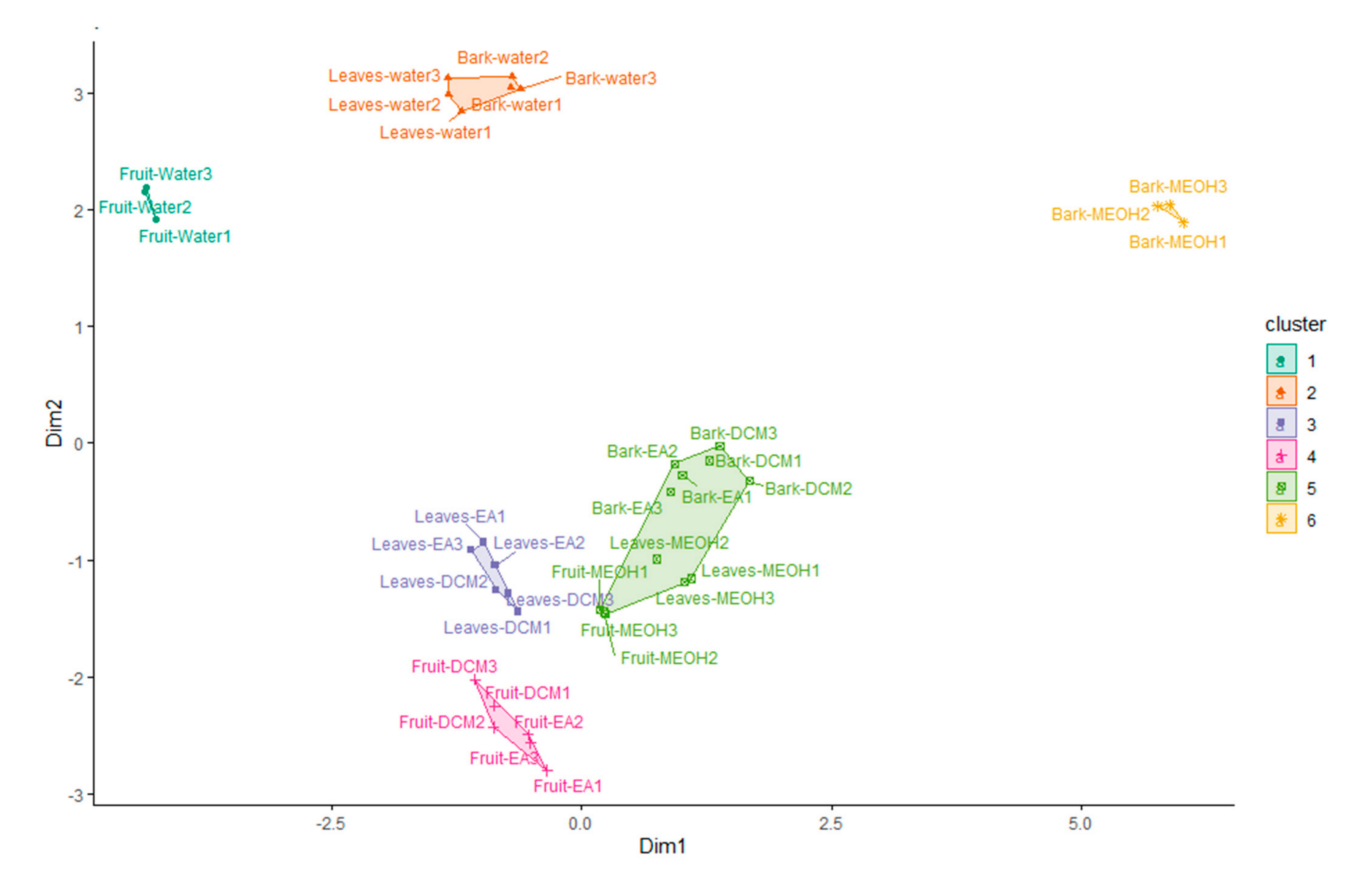
Cluster map of unsupervised hierarchical clustered analysis (similarity: Euclidean, linkage rule: Ward).

**Figure 10:**
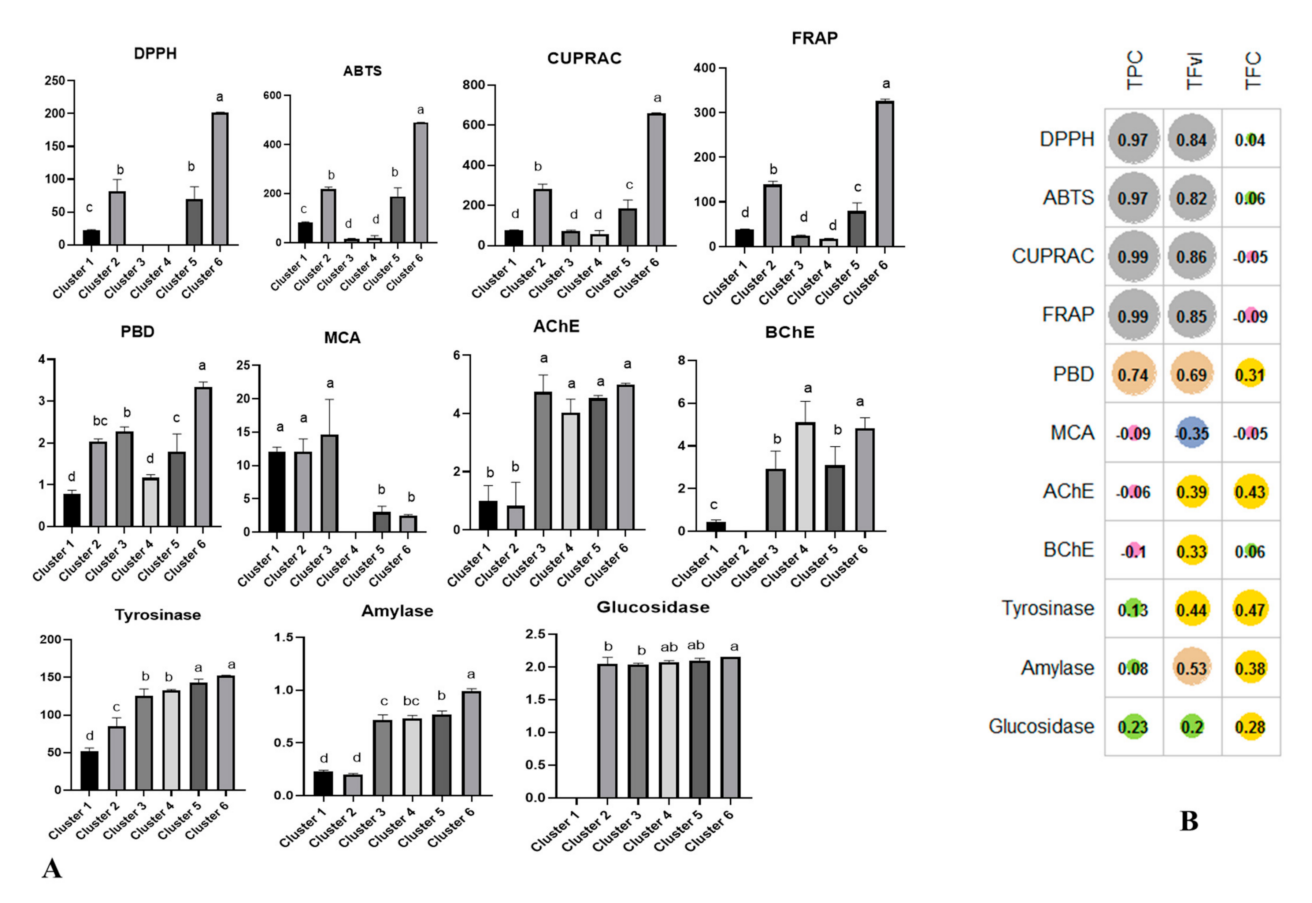
Investigation of biological activities characterizing the different clusters and their relationship with phenolic contents. (**A**), Comparison in obtained clusters; (**B**), Pearson correlation between total bioactive compounds and biological activity assays. Different letters (a–d) indicate significant differences in the clusters.

PCA proved to be very useful since it allowed to show a heterogeneous distribution of *B. retusa* samples in the respective score plots. Nevertheless, it was difficult for us to achieve the typology of some samples including DCM, EA, MEOH extracts of fruit, DCM and EA extracts of bark and MEOH extract of leaves. For this purpose, the result of PCA was submitted to hierarchical clustered analysis. The purpose of this approach is to emphasize clustering of samples according to their similarity/dissimilarity. The cluster map is presented in Figure 9. Six main clusters were obtained among which some were previously identify in the PCA score plots. To highlight the characteristics of the samples constituting the different clusters, the comparison of mean values of each biological activity were reported in Figure 10A. It can be seen, the sample (MEOH-Bark) representative of the cluster 6 was the most active; indeed, it combined a highest antioxidant activity and ability to suppress amylase and glucosidase. Additionally, despite it had lowest Fe^2+^ chelating activity, it showed excellent inhibition against BChE and tyrosinase on an equal footing with the samples constituting the clusters 4 and 5, respectively. Further, the samples of clusters 3, 4, 5 and 6 were found to be more active inhibitor compared with those of the first and second clusters.

A substantial variation was found among the extracts of different parts of *B. retusa*, lengthened due to the variation in bioactive contents of the respective organs. The bark contain considerably more phenolic constituents and methanol were the most efficient extraction solvent in contrast to ethyl acetate, dichloromethane and water. Thus, it can be said that phenolic are not disseminate equally through the organs of *B. retusa*. Additionally, compounds extracted from of *B. retusa* might be polar in nature.

The correlation between the 11 biological activities and the three phenolic contents (TPC, TFC, TFvl) were assessed to clarify their relationships (Figure 10B). The total phenolic content and total flavanol content were positively correlated with antioxidant activities (DPPH, ABTS, FRAP, CUPRAC and PBD); that means that the antioxidant activity of *B. retusa* samples increase with increased phenolic content. As earlier reported bark presented the highest polyphenolic content, in particular, it contained Epicatechin, epigallocatechin, proanthocyanidins known as powerful antioxidant agents. In fact, Pauli et al. [41] reported that the highest antioxidant of *Camellia sinensis* (L.) Kuntze leaves is link to the presence of abundant catechin compounds such as epicatechin and epigallocatechin. The condensed tannin, i.e., proanthocyanidins constituting of catechin and epicatechin was also point out to possess significant antioxidant property [42].

## 3. Materials and Methods

### 3.1. Plant Material and Preparation of Extracts

*Breynia retusa* materials were collected in the neighbourhood of the Banco rainforest in the municipality of Abobo (Abidjan, Ivory Coast) in January 2019 by Kouadio Ibrahime Sinan. The plants were identified by Dr. Kouadio Bene (Université Nangui Abrogoua, Abidjan, Ivory Coast). The plant samples were deposited in the Department of Biology at Selcuk University. The leaves, stem barks and flowers were used. The plant materials were dried in shade for about 10 days and then grounded using a laboratory mill.

Maceration was used for sample preparation with dichloromethane, ethyl acetate and methanol as extraction solvents. For this purpose, 5 g of the samples were macerated for 24 h at room temperature. After this, the mixtures were filtered and then the solvents were removed via a rotary-evaporator. With respect to the water extracts, 5 g of the plant material were infused with 100 mL boiled water. Thereafter, the water was dried using freeze-drying. All extracts were stored at +4 °C until further studies.

### 3.2. Profile of Bioactive Compounds

The total phenolic (TPC), flavanol (TFvl) and flavonoid (TFC) contents of the extracts were measured and detailed methods were described in our previous paper [43]. Standards, namely gallic acid (GAE) for phenolics, catechin (CE) for flavanol and rutin (RE) for flavonoids, were used to explain the results.

#### HPLC-DAD-MS^n^ Analysis, Isolation of Epicatechin-7-*O*-Sulphate

The dried extracts (50 mg) were dissolved in deuterated chloroform (700 microliters) using ultrasound bath for 5 min, then the sample was centrifuged and transferred to NMR tube for measurement. The residue non dissolved was dried under vacuum, then was treated with deuterated methanol, sonicated for 5 min centrifuged and transferred to tube for NMR measurement. Other amount of extract (50 mg) was dispersed in deuterated water sonicated for 5 min, centrifuged and used for the NMR analysis. NMR spectra were obtained on a Bruker Avance III 400 Ultra shielded spectrometer with superconducting 400 MHz magnet system (Bruker, Ettlingen, Germany). NMR spectra was acquired in deuterated chloroform (Sigma-Aldrich, Milan, Italy). Durian^®^ 4.95 mm NMR tubes (Duran Group, Mainz, Germany) were utilized. Chemical shifts are expressed in δ values.

Dried extracts were exactly weighted—15 mg—and dissolved in 1.5 mL of ethanol and sonicated for 20 min. The extracts were then centrifuged at 13,000 rpm and used for analysis. Extract was analyzed using Electrospray (ESI) source. The instrumentation was Agilent 1260 chromatograph (Santa Clara, CA, USA) equipped with 1260 diode array detector (DAD) and Varian MS-500 ion trap mass spectrometer (Varian inc, Palo Alto, CA, USA) equipped with ESI source (Varian inc, Palo Alto, CA, USA). At the end of the column, two “T” splitters separated the flow rate: half of the liquid was split to DAD and half to Agilent/Varian MS-500 ion trap mass spectrometer. Ultraviolet-visible (UV-Vis) spectra (Varian inc, Palo Alto, CA, USA) were acquired in the range of 190–400 nm.

Three different methods were performed for the proanthocyanidins and spiroketal glycosides analysis. First, an Agilent Eclipse XDB C-18 (3.0 × 150 mm) 3.5 μm was used as stationary phase and as eluents water 0.1% formic acid (A), acetonitrile (B) were used. Elution gradient started with 90% of A and 10% of B and go to 100% of B in 30 min. The flow rate was 1.00 mL/min. Second, an Agilent Zorbax SB-C3 (4.6 × 150 mm, Agilent, Santa Clara, CA, USA) 5 μm coupled with an Agilent Eclipse XDB C-18 (3.0 × 150 mm, Agilent, Santa Clara, CA, USA) 3.5 μm were used as stationary phase to increase the number of plates. The same eluents and elution gradient were employed. Third, the same two columns were used as stationary phase and as eluents, water 0.1% formic acid (A), acetonitrile (B) and methanol (C) were used. Elution gradient started with 98% of A and 2% of B and go to 35% of A, 60% of B and 5% of C in 120 min. Then the amount of B was increased to 90% while the amount of C was increased to 10% in 80 min. The flow rate was 400 µL/min.

The MS spectra were acquired in negative ion mode in 50–2000 Da range, using an ESI source, drying gas temperature was initially 350 °C and in 20 min decrease to 300, drying gas pressure was 35 psi, nebulizer was 35 psi, needle was set at 4500 V, RF 85% and capillary 80 V. Varian MS 500 mass spectrometer was used, using ESI as ion source in order to identify proanthocyanidins and spiroketal glycosides on the base of MSn spectra. Quantification of compounds was obtained using DAD. Catechin was used for the quantification of proanthocyanidins using solutions (range 180–1 µg/mL) at 280 nm and calibration curve was Y = 31.62X +1.021 (R2 = 0.9993).

For the isolation of epicatechin-7-*O*-sulphate 5 g of dried methanol extract was applied to a sephadex column (4 × 100 cm) and eluted with methanol 0,5 mL/min, using a Dionex P680 pump. Fraction of 10 mL were collected using a Varian 920 fraction collector, 300 fractins obtained and pooled on the basis of their TLC behaviour (Solvent Dichloromethane: Methanol: Water: Acetic acid 10:6:0,5:1) in 20 groups. Fraction 96–113 (650 mg) was used for semipreparative HPLC. Agilent 1100 system equipped with diode array and fraction collector was used, Agilent XDB C-18 3 × 150 mm was used as stationary phase. Flow rate was 5 mL/min and solvent was isocratic mixture of Acetonitrile (45) water 1% formic acid (55). Peak was collected on the basis of absorbance at 280 nm. Epicatechin-7-*O*-sulphate was obtained as dark yellow powder (50 mg) and structure was elucidated on the basis of its NMR and MS data. NMR assignments are reported in Table 6

### 3.3. Determination of Antioxidant and Enzyme Inhibitory Effects

To detect antioxidant properties, we used several chemical assays with different mechanisms namely, radical scavenging, reducing power and metal chelating. Trolox (TE) and ethylenediaminetetraacetic acid (EDTA) were used as standard antioxidant compounds. Obtained results were expressed as equivalents of these compounds [44]. To detect inhibitory effects on enzymes, we used colorimetric enzyme inhibition assays and these assays included tyrosinase, α-glucosidase, α-amylase and cholinesterases. Some standard inhibitors (galantamine, kojic acid and acarbose) were used as positive controls.

### 3.4. Statistical Analysis

All tests were repeated three times and results were done as means following by the standard deviation. The means values comparisons were done by two way-ANOVA following by Tukey’s post-hoc test at the 5% level of significance using Xlstat software version 2018 (Addinsoft Corporation). Multivariate analysis (Principal Component Analysis (PCA) and Hierarchical Clustered Analysis) were achieved using R software version 3.6.1. Before performing PCA, all variables were auto scaled. The selection of the components was relied on two metrics; the components having an eigenvalue above 1 and components which the cumulative percentage of variance explained was equal to or more than 80%. Finally, the correlation coefficient between the phenolic contents (TPC, TFC, TFvl) and the biological activities were calculated.

## 4. Conclusions

In conclusion, the methanolic bark extract of *B. retusa* yielding the highest phenolic and flavanol contents demonstrated substantial in vitro antioxidant potential with all assays, except with metal chelating assay, whereby the ethyl acetate leaf extract was most active. The observed antioxidant activity might be caused by the synergistic action of bioactive compounds present in the extracts. The methanolic bark extract significantly depressed tyrosinase, α-amylase and α-glucosidase activity. LC-DAD-MS^n^ analysis revealed the presence of numerous procyanidin derivatives in bark as well as catechins and gallotannins. All these compounds are well known for their antioxidant properties. Furthermore, breynin, spiroketal sulfur containing glycosides were detected in barks. Fruits and leaves mostly contained flavonoid glycosides and procyanidins and breynin were not detected in these latter two extracts. This research work has presented valuable primary data on *B. retusa*, but need further investigations, such as in vivo studies, bioavailability and toxicity before projecting this plant for future nutraceutical/functional food and/or for possible pharmaceutical applications.

## Figures and Tables

**Table 1:** Total bioactive compounds and total antioxidant capacity (by phosphomolybdenum assay) of the studied extracts.

Parts	Solvents	Total Phenolic Content (mg GAE/g)	Total Flavonoid Content (mg RE/g)	Total Flavanol Content (mg CE/g)	Phosphomolybdenum (mmol TE/g)
Bark	DCM	63.54 ± 1.23 ^d^	24.13 ± 0.90 ^b^	11.59 ± 0.18 ^b^	2.19 ± 0.22 ^bc^
	EA	49.43 ± 0.42 ^e^	24.27 ± 0.38 ^b^	9.26 ± 0.18 ^c^	1.89 ± 0.23 ^c^
	MeOH	145.79 ± 0.84 ^a^	4.79 ± 0.21 ^f^	35.24 ± 0.39 ^a^	3.33 ± 0.13 ^a^
	Water	75.28 ± 0.65 ^c^	1.19 ± 0.20 ^hi^	1.25 ± 0.02 ^g^	2.08 ± 0.06 ^bc^
Fruit	DCM	18.23 ± 0.13 ^j^	2.80 ± 0.08 ^g^	2.30 ± 0.02 ^e^	1.15 ± 0.11 ^de^
	EA	14.17 ± 0.33 ^k^	2.66 ± 0.10 ^g^	1.80 ± 0.03 ^f^	1.19 ± 0.04 ^d^
	MeOH	37.52 ± 0.27 ^g^	2.28 ± 0.03 ^gh^	2.94 ± 0.08 ^d^	1.17 ± 0.02 ^d^
	Water	26.00 ± 0.32 ^h^	0.64 ± 0.05 ^i^	0.70 ± 0.01 ^h^	0.79 ± 0.08 ^e^
Leaf	DCM	24.51 ± 0.17 ^h^	11.79 ± 0.36 ^d^	2.01 ± 0.01 ^ef^	2.27 ± 0.09 ^b^
	EA	21.63 ± 0.69 ^i^	19.18 ± 0.76 ^c^	1.74 ± 0.01 ^f^	2.29 ± 0.14 ^b^
	MeOH	47.59 ± 0.66 ^f^	34.49 ± 0.18 ^a^	1.84 ± 0.02 ^f^	1.93 ± 0.16 ^bc^
	Water	77.63 ± 0.63 ^b^	7.28 ± 0.51 ^e^	0.74 ± 0.01 ^h^	2.00 ± 0.02 ^bc^

Values expressed are means ± S.D. of three parallel measurements. GAE: Gallic acid equivalent; RE: Rutin equivalent; TE: Trolox equivalent. CE: Catechin equivalent; DCM: Dichloromethane; EA: Ethyl acetate; MeOH: Methanol. Different letters of superscripts indicated significant differences for each extract (*p* < 0.05).

**Table 2:** Quantification of compound detected by LC-MS in *B. retusa* stem barks.

	Retention Time	*m*/*z*	Fragments	Identification	mg/g
			**Catechins**		
E1	31.7	385	305 (100) 287 (15) 195 (3) 161 (3)	epigallocatechin sulphate	1.61 ± 0.05
E2	34.9	369.5	289 (100) 245 (24) 217 (52)	Epicatechin-7-*O*-sulphate	60.53 ± 0.35
E3	35.2	305	287 (15) 195 (3) 161 (3)	Epigallocatechin	0.46 ± 0.05
E4	38.7	289	245 (100) 205 (52) 175 (20)	Epicatechin	4.89 ± 0.08
				total amounts	67.48 ± 0.08
			Proanthocyanidins		
D1	30.9	577.5	451 (28) 425 (100) 408 (15) 289 (30) 245 (6)	PAC-B dimer	0.81 ± 0.02
D2	31.9	577.5	451 (26) 425 (100) 408 (15) 289 (30) 245 (6)	PAC-B dimer	0.97 ± 0.04
D3	35.8	577.5	451 (29) 425 (100) 408 (17) 289 (35) 245 (9)	PAC-B dimer *	6.68 ± 0.21
D4	37.9	577.5	451 (28) 425 (100) 408 (15) 289 (27) 245 (8)	PAC-B2	13.07 ± 0.51
D5	47.8	577.5	451 (31) 425 (100) 408 (18) 289 (26) 245 (10)	PAC-B dimer *	2.21 ± 0.10
T1	34.4	865	739 (14) 713 (24) 695 (100) 577 (28) 575 (21) 543 (9) 425 (8) 407(8)	PAC-B trimer	1.17 ± 0.07
T2	39.3	865	739 (14) 713 (24) 695 (100) 577 (28) 575 (21) 543 (9) 425 (8) 407(8)	PAC-B trimer	4.06 ± 0.55
T3	40.7	865	739 (17) 713 (29) 695 (100) 577 (33) 575 (16) 543 (9) 425 (10) 407(8)	PAC-B trimer	1.39 ± 0.05
T4	43.6	865	739 (11) 713 (30) 695 (100) 577 (22) 575 (23) 543 (11) 425 (8) 407(11)	PAC-B trimer *	14.57 ± 0.57
T5	45.4	865	739 (14) 713 (24) 695 (100) 577 (28) 575 (21) 543 (9) 425 (8) 407(8)	PAC-B trimer	1.61 ± 0.05
T6	49.7	865	739 (16) 713 (21) 695 (100) 577 (25) 575 (22) 543 (12) 425 (11) 407(8)	PAC-B trimer	1.32 ± 0.07
TT1	41.0	1154.5	1028 (78) 1002 (40) 984 (100) 865 (58) 739 (51) 577 (39)	PAC-B tetramer	1.76 ± 0.06
TT2	46.1	1154.5	1028 (68) 1002 (45) 984 (100) 865 (63) 739 (53) 577 (39)	PAC-B tetramer	6.09 ± 0.15
TT3	46.6	1154.5	1028 (66) 1002 (43) 984 (100) 865 (60) 739 (50) 577 (39)	PAC-B tetramer	1.43 ± 0.05
TT4	50.5	1154.5	1028 (70) 1002 (44) 984 (100) 865 (62) 739 (53) 577 (39)	PAC-B tetramer	1.47 ± 0.05
HX	48.6	865 *	1441 (25) 1151 (100) 989 (20) 713 (24) 695 (100) 577 (5)	PAC-B hexamer	1.28 ± 0.07
HP	49.8	1009 *	1441 (20) 1151 (100) 989 (22) 713 (26) 695 (100) 577 (3)	PAC-B heptamer	3.73 ± 0.15
				total amounts	63.63 ± 0.11
			**Small Phenolics**		
	13.01	331	271 169 151	galloylglucose	0.21 ± 0.’02
	20.1	331	271 169 151	galloylglucose	0.23 ± 0.01
	20.9	315	153	Protocatechuic acid-*O*-hexoside	0.95 ± 0.03
	31.3	401	293 269 161	benzoyl alchol hexose pentose	0.60 ± 0.02
				total amounts	1.99 ± 0.04
			**Spiroketal Glycosides**		
	44.8	952	813 463 368	Breinin B	
	47.6	936	798 472 453	Breinin D	
	51.9	952	813 463 368	Epibreinin B	
	56.9	922	790 464 353	Breinin F derivative no sulfoxide	

* Double charged ions [M − 2H]^2−^.

**Table 3:** Quantification of compound detected by LC-DAD-MS^n^ in *B. retusa* leaves and fruit.

					Leaves	Fruits
	rt	[M − H]^−^	Fragments	Identification	mg/g	mg/g
1	5.6	377	342 202	Disaccharide (chlorine adduct)	Not quantified	Not quantified
2	5.78	376	341 202	Disaccharide (chlorine adduct)	Not quantified	Not quantified
3	5.97	195	177 159 129	Gluconic acid	Not quantified	Not quantified
4	6.2	341	179	caffeoyl hexose	0.84 ± 0.01	2.37 ± 0.01
5	8.55	290	272 254 200 128	Unknown	nd	nd
6	31.0	401	293 269 161	benzoyl alchol hexose pentose	3.88 ± 0.01	1.99 ± 0.01
7	33.4	423,5	313 169 125	Galloyl hexose derivative	1.01 ± 0.01	11.18 ± 0.01
8	45.2	625	301 272 255	quercetin di hexoside	16.37 ± 0.01	1.89 ± 0.01
9	49.1	609	448 285	Kaempferol-*O*-hexoside-*O*-hexoside	5.43 ± 0.01	1.59 ± 0.01
10	51.5	463	301 255 179	Quercetin-3-*O*-glucoside	11.92 ± 0.01	11.16 ± 0.01
11	54.1	187	129 97	Gallic acid monohydrate	0.30 ± 0.01	1.37 ± 0.01
12	39.0	449	311 287 269 259	Dihydrokaempferol hexoside	1.37 ± 0.01	nd
13	34.8	595	505 475 415 385 355	Naringenin-6,8-di-*C*-glucoside	3.35 ± 0.01	nd
14	50.0	421	405 289 251 221 178	Epicatechin/catechin pentoside	2.47 ± 0.01	nd
15	55.9	447	284 255 227	Kaempferol-7-*O*-hexoside	4.31 ± 0.01	nd
				Total amount	51.27 ± 0.02	31.55 ± 0.02

**Table 4:** Antioxidant properties of the tested extracts.

Parts	Solvents	DPPH (mg TE/g)	ABTS (mg TE/g)	CUPRAC (mg TE/g)	FRAP (mg TE/g)	Metal Chelating Activity (mg EDTAE/g)
Bark	DCM	93.53 ± 0.17 ^c^	231.93 ± 1.38 ^b^	227.75 ± 5.92 ^d^	103.77 ± 2.29 ^d^	2.40 ± 0.87 ^de^
	EA	80.43 ± 0.69 ^d^	204.34 ± 1.82 ^c^	215.33 ± 1.15 ^e^	87.40 ± 1.35 ^e^	2.49 ± 0.73 ^de^
	MeOH	202.09 ± 0.15 ^a^	490.12 ± 0.18 ^a^	661.82 ± 0.40 ^a^	325.86 ± 4.36 ^a^	2.50 ± 0.13 ^de^
	Water	98.09 ± 0.36 ^b^	225.38 ± 3.91 ^b^	304.88 ± 1.16 ^b^	145.36 ± 0.82 ^b^	10.84 ± 0.73 ^bc^
Fruit	DCM	Na	7.23 ± 1.33 ^i^	43.32 ± 0.26 ^i^	16.44 ± 0.11 ^j^	Na
	EA	Na	27.53 ± 1.32 ^g^	73.16 ± 4.20 ^h^	18.06 ± 0.51 ^j^	Na
	MeOH	48.43 ± 0.82 ^g^	142.74 ± 2.53 ^e^	127.35 ± 3.25 ^g^	59.90 ± 0.73 ^g^	3.82 ± 0.67 ^d^
	Water	21.72 ± 1.44 ^h^	83.25 ± 1.98 ^f^	77.66 ± 0.69 ^h^	38.45 ± 0.61 ^h^	12.03 ± 0.72 ^bc^
Leaf	DCM	na	14.95 ± 1.15 ^h^	72.98 ± 1.69 ^h^	24.07 ± 0.63 ^i^	9.88 ± 1.75 ^c^
	EA	na	17.02 ± 0.99 ^h^	76.27 ± 1.40 ^h^	25.29 ± 0.42 ^i^	19.34 ± 0.31 ^a^
	MeOH	56.94 ± 0.74 ^f^	176.18 ± 3.71 ^d^	174.75 ± 1.85 ^f^	71.77 ± 1.92 ^f^	3.41 ± 0.56 ^d^
	Water	66.15 ± 3.51 ^e^	209.79 ± 4.50 ^c^	259.48 ± 2.61 ^c^	132.85 ± 1.31 ^c^	13.35 ± 1.94 ^b^

Values expressed are means ± S.D. of three parallel measurements. TE: Trolox equivalent; EDTAE: EDTA equivalent. DCM: Dichloromethane; EA: Ethyl acetate; MeOH: Methanol; na: not active. Different letters of superscripts indicated significant differences for each extract (*p* < 0.05).

**Table 5:** Enzyme inhibitory properties of the tested extracts.

Parts	Solvents	AChE Inhibition (mg GALAE/g)	BChE Inhibition (mg GALAE/g)	Tyrosinase Inhibition (mg KAE/g)	α-Amylase Inhibition (mmol ACAE/g)	α-Glucosidase Inhibition (mmol ACAE/g)
Bark	DCM	4.55 ± 0.08 ^ab^	2.69 ± 0.86 ^cde^	143.23 ± 0.60 ^b^	0.74 ± 0.03 ^cd^	2.06 ± 0.01 ^c^
	EA	4.57 ± 0.14 ^ab^	2.13 ± 0.16 ^e^	135.76 ± 0.55 ^c^	0.76 ± 0.02 ^bcd^	2.07 ± 0.01 ^c^
	MeOH	5.00 ± 0.03 ^ab^	4.82 ± 0.50 ^ab^	152.79 ± 0.27 ^a^	0.99 ± 0.01 ^a^	2.16 ± 0.01 ^a^
	Water	1.55 ± 0.17 ^d^	na	75.51 ± 0.56 ^f^	0.21 ± 0.01 ^e^	2.14 ± 0.01 ^a^
Fruit	DCM	3.64 ± 0.32 ^c^	4.43 ± 0.47 ^ab^	132.04 ± 2.57 ^c^	0.72 ± 0.01 ^cd^	2.06 ± 0.01 ^c^
	EA	4.40 ± 0.08 ^abc^	5.77 ± 0.94 ^a^	132.79 ± 0.72 ^c^	0.75 ± 0.03 ^bcd^	2.10 ± 0.01 ^b^
	MeOH	4.55 ± 0.05 ^ab^	3.65 ± 0.03 ^bcd^	145.34 ± 0.13 ^b^	0.77 ± 0.01 ^bc^	2.14 ± 0.01 ^a^
	Water	0.98 ± 0.54 ^d^	0.46 ± 0.09 ^f^	52.44 ± 3.87 ^g^	0.23 ± 0.01 ^e^	na
Leaf	DCM	4.33 ± 0.61 ^bc^	3.58 ± 0.24 ^bcde^	117.92 ± 4.12 ^d^	0.75 ± 0.04 ^cd^	2.06 ± 0.01 ^cd^
	EA	5.13 ± 0.11 ^a^	2.25 ± 0.62 ^de^	133.75 ± 0.54 ^c^	0.69 ± 0.03 ^d^	2.03 ± 0.01 ^d^
	MeOH	4.41 ± 0.05 ^abc^	3.89 ± 0.67 ^bc^	147.68 ± 0.53 ^ab^	0.82 ± 0.02 ^b^	2.12 ± 0.01 ^b^
	Water	0.09 ± 0.01 ^e^	na	95.50 ± 3.24 ^e^	0.19 ± 0.01 ^e^	1.97 ± 0.02 ^e^

Values expressed are means ± S.D. of three parallel measurements. GALAE: Galantamine equivalent; KAE: Kojic acid equivalent; ACAE: Acarbose equivalent; DCM: Dichloromethane; EA: Ethyl acetate; MeOH: Methanol; na: not active. Different letters of superscripts indicated significant differences for each extract (*p* < 0.05).

**Table 6:** NMR assignments for Epicatechin-7-*O*-sulphate.

Position	δ H	δ C
2	4.90 (under methanol)	78.0
3	4.25 br s (*J* = 1.6)	65.9
4	2.97–3.03	27.9
5	-	157.8
6	6.23 (*J* = 1.6)	98.9
7	-	156.4
8	6.64 (*J* = 1.6)	101.8
9	-	151.4
10	-	104.0
